# Reference ranges and influencing factors of pressure pain threshold in Chinese patients with knee osteoarthritis

**DOI:** 10.1080/07853890.2026.2664304

**Published:** 2026-05-11

**Authors:** Zhiheng Hu, Xiaofeng Chang, Shuxin Yao, Feng Ye, Chao Xu, Jianbing Ma

**Affiliations:** ^a^ Department of Knee Joint Surgery, Honghui Hospital, Xi’an Jiaotong University, Xi’an, Shaanxi Province, China; ^b^ The Graduate School of Xi’an Medical University, Xi’an, Shaanxi Province, China; ^c^ Department of Health Statistics, Faculty of Preventive Medicine, the Air Force Military Medical University, Xi’an, Shaanxi Province, China

**Keywords:** Knee osteoarthritis, pressure pain threshold, reference ranges, central sensitization, central sensitization inventory, total knee arthroplasty

## Abstract

**Background:**

Pressure pain threshold (PPT) is a reliable objective measure of pain sensitization; however, standardized region-specific reference data for Chinese patients with knee osteoarthritis (KOA) remain scarce. This study aimed to establish preliminary PPT reference ranges for end-stage KOA patients and age- and sex-matched healthy controls in Xi’an, Northwest China, and to identify factors influencing PPT.

**Methods:**

A total of 165 patients with end-stage KOA scheduled for total knee arthroplasty and 146 age- and sex-matched healthy controls were enrolled. PPT at the medial knee and dorsal forearm was assessed using standardized PPT assessment. Demographic characteristics, body mass index (BMI), sociological factors and Central Sensitization Inventory (CSI) scores were collected. Nonparametric analyses, Spearman correlation and hierarchical regression were performed.

**Results:**

KOA patients exhibited significantly lower median PPTs (forearm: 3.79; knee: 4.21 kg·cm^−2^) compared with controls (forearm: 5.53; knee: 6.57 kg·cm^−2^; all *p* < 0.001), with left-shifted and broader 95% reference ranges (KOA: forearm 2.16–6.09; knee 2.07–6.78 kg·cm^−2^). CSI scores were moderately negatively correlated with forearm PPT (r = −0.567) and weakly correlated with knee PPT (r = −0.389; all *p* < 0.001). After adjusting for confounders, CSI remained the strongest independent predictor of PPT (forearm: β = −0.403; knee: β = −0.301; all *p* < 0.001), explaining greater incremental variance (ΔR^2^ = 0.144 for forearm; ΔR^2^ = 0.080 for knee) than other factors. In healthy controls, PPT was influenced only by sex, age and BMI (all *p* < 0.01).

**Conclusions:**

This study is the first to establish preliminary PPT reference ranges for end-stage KOA patients in Xi’an, Northwest China, suggesting generalized pain hypersensitivity as a predominant phenotype in this cohort. The strong correlation between CSI (subjective) and forearm PPT (objective) provides a preliminary basis for future preoperative stratification and potential perioperative analgesic strategies, with potential clinical translational value.

## 1. Introduction

Knee osteoarthritis (KOA) is one of the most prevalent degenerative joint diseases in clinical practice. Chronic pain and functional impairment associated with KOA not only markedly reduce patients’ quality of life but also impose substantial socioeconomic and healthcare burdens [1–3]. As the primary clinical manifestation of KOA, pain exhibits considerable heterogeneity in its pathogenesis. Beyond local structural lesions such as articular cartilage degradation and osteophyte formation, inflammation-mediated peripheral sensitization and central sensitization (CS) have been identified as key neuropathological mechanisms underlying sustained amplification of pain signals, disease chronicity and pain spreading beyond the affected joint [2,4–6].

Pressure pain threshold (PPT) assessment is an internationally recognized tool for evaluating pain processing function. PPT quantifies the tolerance to noxious mechanical stimuli, comprehensively reflecting peripheral nociceptor sensitivity, spinal pain signal integration and cortical descending inhibitory control – thus serving as a core objective indicator linking peripheral and CS [4,5,7]. Numerous studies have confirmed that PPT demonstrates excellent reliability and validity in KOA populations [8–12]. Importantly, the correlation between PPT and subjective pain intensity in KOA patients is stronger than that between PPT and radiographic structural damage [13–15]. This suggests that PPT can help overcome the clinical dilemma of ‘discordance between radiographic damage and pain severity’ and more accurately capture the neurobiological basis of pain.

The Central Sensitization Inventory (CSI) is a standardized self-report instrument designed to assess CS-related symptoms, including widespread pain, tactile allodynia and associated emotional and cognitive burdens. Renowned for its simplicity and efficiency, the CSI has been widely applied in various chronic pain conditions, such as fibromyalgia and chronic low back pain [16]. Our previous research confirmed that the cross-culturally adapted simplified Chinese version of the CSI exhibits excellent reliability and validity in Chinese KOA patients [17], providing a valuable tool for CS assessment in this population.

From an assessment perspective, PPT serves as an objective physiological indicator quantifying pain function, whereas the CSI captures subjective symptom experiences. Together, they provide complementary evaluations of CS, encompassing objective functional quantification and individual symptom perception, theoretically enabling synergistic application. However, existing studies have reported inconsistent findings regarding their correlation [18,19], suggesting that the relationship may be influenced by disease subtype heterogeneity and confounding factors.

Currently, two critical gaps exist in KOA pain assessment in China. First, although international studies have reported PPT reference ranges for multiple anatomical sites and collected substantial disease-specific data [20–22], pain sensitivity is modulated by ethnic, genetic, cultural and lifestyle factors [23–25]. Consequently, these data cannot be directly extrapolated to Chinese populations across diverse geographic regions, and region-specific PPT reference ranges for the knee (local site) and forearm (remote site) in end-stage KOA patients in Northwest China remain unestablished. Second, existing studies have not systematically adjusted for sociodemographic confounders (body mass index [BMI], educational level, living alone status) in multivariable models, nor clarified the independent predictive value of PPT and CSI for KOA pain assessment. This limits the clinical utility of their combined application and hinders precise identification of KOA sensitization subtypes using PPT alone.

To address these gaps, the present study applied an internationally standardized PPT assessment to measure PPT at the knee and forearm in end-stage KOA patients in Xi’an, Northwest China, and age- and sex-matched healthy controls. The primary objective was to establish the first set of preliminary region-specific PPT reference ranges for end-stage KOA patients in Xi’an, Northwest China. Additionally, Spearman correlation analysis and hierarchical multiple linear regression were used to delineate the relationship between CSI and PPT and to evaluate their independent predictive values within a multifactorial framework. The findings of this study aim to (1) fill the gap in region-specific PPT reference ranges for end-stage KOA patients in Northwest China, (2) clarify the core correlation and independent predictive value of CSI and PPT, and (3) provide preliminary objective evidence for assessing pain sensitivity and exploring potential associations between PPT and CS in end-stage KOA patients. These findings may inform future investigations of KOA pain mechanisms and stratified pain management, and guide optimization of the combined ‘CSI + PPT’ assessment model to improve clinical pain evaluation. The wider applicability of these results should be validated in multi-centre studies across China.

## 2. Methods

### 2.1. Sample size estimation

The KOA group served as the primary analysis population, with data collected in a single session to eliminate dropout concerns. Sample size was estimated using G*Power 3.1.9.7, considering both correlation analyses and hierarchical regression with six independent variables. Based on a pilot study (*n* = 20) showing moderate negative correlations between CSI and forearm/knee PPT (r ≈ −0.50/–0.35), and setting α = 0.05 and power = 0.90, minimum sample sizes of 36 and 72 participants were calculated for the KOA group. Following the regression rule of thumb (sample size ≥ 10 × number of independent variables), 120 participants were planned for the KOA group, with 165 ultimately enrolled. A total of 146 healthy controls were recruited to ensure stable group comparisons. Post-hoc power analysis confirmed a statistical power of 0.992 for the KOA group.

### 2.2. Participants

This was a cross-sectional case-control study conducted from January 2025 to October 2025, enrolling a total of 311 participants. The experimental group consisted of 165 patients with end-stage KOA scheduled for unilateral total knee arthroplasty (TKA), all of whom met the diagnostic criteria of the American College of Rheumatology (ACR) [26] and had a Kellgren-Lawrence (KL) grade of IV on X-ray [27]. The control group included 146 age- and sex-matched healthy participants with no history of chronic pain such as osteoarthritis or rheumatoid arthritis, and an average pain score ≤ 3 points in the past 4 weeks.

The exclusion criteria were identical for both groups and included: use of analgesics, nonsteroidal anti-inflammatory drugs, or sedative medications within 48 h before assessment; skin damage, infection, obvious scarring, or a history of fracture at the measurement sites; acute illness or recent trauma; major chronic systemic diseases, including severe cardiovascular disease, diabetes mellitus, hepatic or renal dysfunction and central or peripheral nervous system disorders; as well as psychiatric disorders, cognitive impairment, or communication difficulties [20–22].

### 2.3. Ethical approval and informed consent

The study was conducted in accordance with the Declaration of Helsinki and was approved by the Medical Ethics Committee of Xi’an Honghui Hospital (Approval No.: 2025-KY-003-01). All participants provided written informed consent after receiving detailed information about the study and were informed of their right to withdraw at any time.

### 2.4. Instrument for PPT assessment

PPT was assessed using the Force Ten^™^ digital pressure algometer (Model FPX 25, indenter area: 1 cm^2^; Wagner Instruments, USA), which has demonstrated excellent reliability, validity and repeatability in previous studies. Measurements using this device exhibit high intra-rater and inter-rater reliability [20,28].

### 2.5. Pain and CS assessment

All assessments were completed within 1 h prior to PPT measurement to avoid mutual interference.

1. Pain intensity: The 11-point Numerical Rating Scale (NRS) was used to evaluate ‘average knee pain intensity over the past 4 weeks’.
2. CS: The simplified Chinese version of the CSI was administered. This 25-item scale is scored on a 5-point Likert scale (0–4 points), yielding a total score of 0–100, with higher scores indicating more prominent CS-related symptoms.

### 2.6. Measurement sites

**Figure 1.**
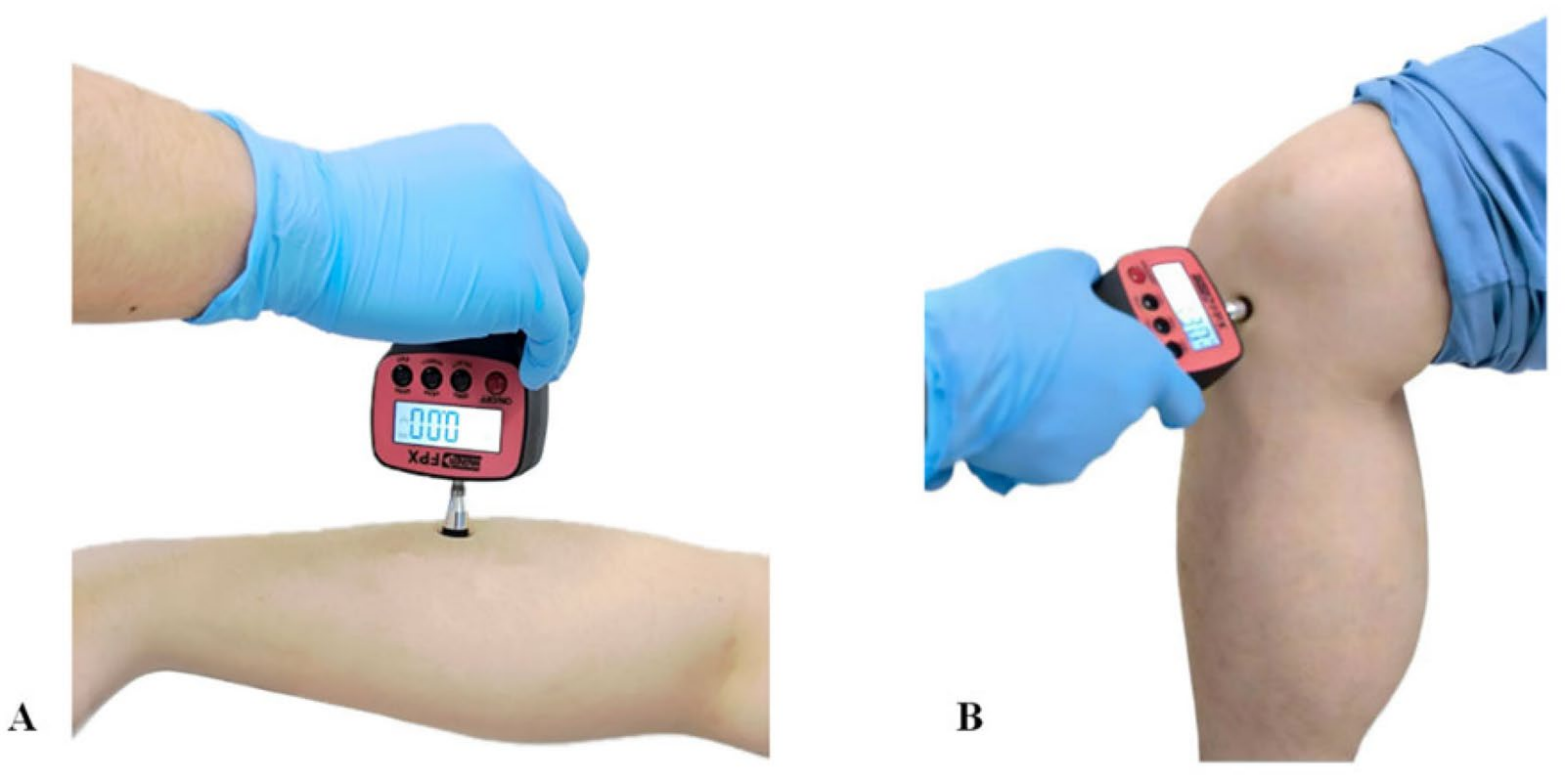
Photographs of PPT measurement sites. (A) PPT measurement at the dorsal forearm; (B) PPT measurement at the medial knee joint.

PPT was measured at two standardized anatomical sites to ensure reproducibility (Figure 1):

1. Medial knee joint: Located on the medial knee joint space line, 3 cm from the midpoint of the medial patellar margin [20,29,30].
2. Dorsal forearm: Located at the midpoint of the line connecting the radial styloid process and lateral epicondyle of the humerus [29,30].

In the KOA group, the affected knee was selected to assess peripheral sensitization, while the contralateral (non-surgical) forearm was used to reflect CS. In healthy controls, either side of the knee and forearm was selected randomly using a random number table method [29,31–33].

### 2.7. PPT assessment protocol

All tests were conducted in a quiet, temperature-controlled room (22–24 °C) by a single researcher who was trained in standardized procedures and blinded to group allocation [34]. Prior to formal measurement, the procedure was demonstrated on a non-measurement area of the participant’s forearm (ventral forearm) to ensure understanding of the instruction: ‘verbally indicate the first perception of pain’. Vertical pressure was applied at a constant rate of 0.5 kg/(cm^2^·s). Pressure was immediately stopped when the participant reported the first sensation of pain, and the corresponding pressure value was recorded as the PPT. Each site was measured three times, with a 30-second interval to prevent local tissue fatigue [20–22]. The average of three measurements was calculated as the final PPT for each site and used in subsequent analyses.

### 2.8. Statistical analysis

Normality of continuous variables was tested using the Kolmogorov–Smirnov test. Non-normally distributed variables were reported as median (interquartile range, IQR), and categorical variables as counts (percentages). Intergroup comparisons were performed using appropriate methods based on variable type: Mann–Whitney U test for continuous variables between two groups, Kruskal–Wallis H test for continuous variables across ≥3 groups, and chi-square test for categorical variables. Ninety-five percent reference ranges for PPT were calculated using the nonparametric percentile method (P_2_._5_–P_97_._5_) [20]. These percentile-based reference ranges were used to describe the distribution of PPT values in the study sample rather than to establish normative reference intervals.

Spearman’s rank correlation was used to assess associations, considering data distribution. Correlation strength (|r|) was classified as negligible (<0.20), weak (0.20–0.39), moderate (0.40–0.59), strong (0.60–0.79), or very strong (≥0.80) [35,36].

Hierarchical multiple linear regression was employed to identify independent predictors of PPT. Given the cross-sectional design, the regression analysis was intended to explore statistical associations rather than infer causal relationships. Variables were entered in blocks: Model 1 included demographic factors (sex, age); Model 2 added BMI; Model 3 included sociological factors (education level, living status); and Model 4 incorporated CSI scores. Categorical variables included in the regression models were coded as dummy variables: sex (male = 0, female = 1, with male as the reference group); living status (not living alone = 0, living alone = 1, with not living alone as the reference group); education level (low = 0, medium/high = 1, with low education level as the reference group; medium and high levels were combined to improve clinical interpretability and ensure adequate sample size for analysis). Model fit was evaluated using adjusted R^2^, and incremental explanatory power by ΔR^2^ and F-test. Multicollinearity was assessed *via* variance inflation factor (VIF < 10), and residual independence by the Durbin–Watson statistic. In the control group, multiple linear regression was performed including all predictors simultaneously. Statistical analyses were conducted using IBM SPSS Statistics 26.0 (IBM Corp., Armonk, NY, USA). Figures were generated using GraphPad Prism 10.0 (GraphPad Software, San Diego, CA, USA). A two-tailed α = 0.05 was considered statistically significant.

## 3. Results

### 3.1. Baseline characteristics of the study population

**Figure 2.**
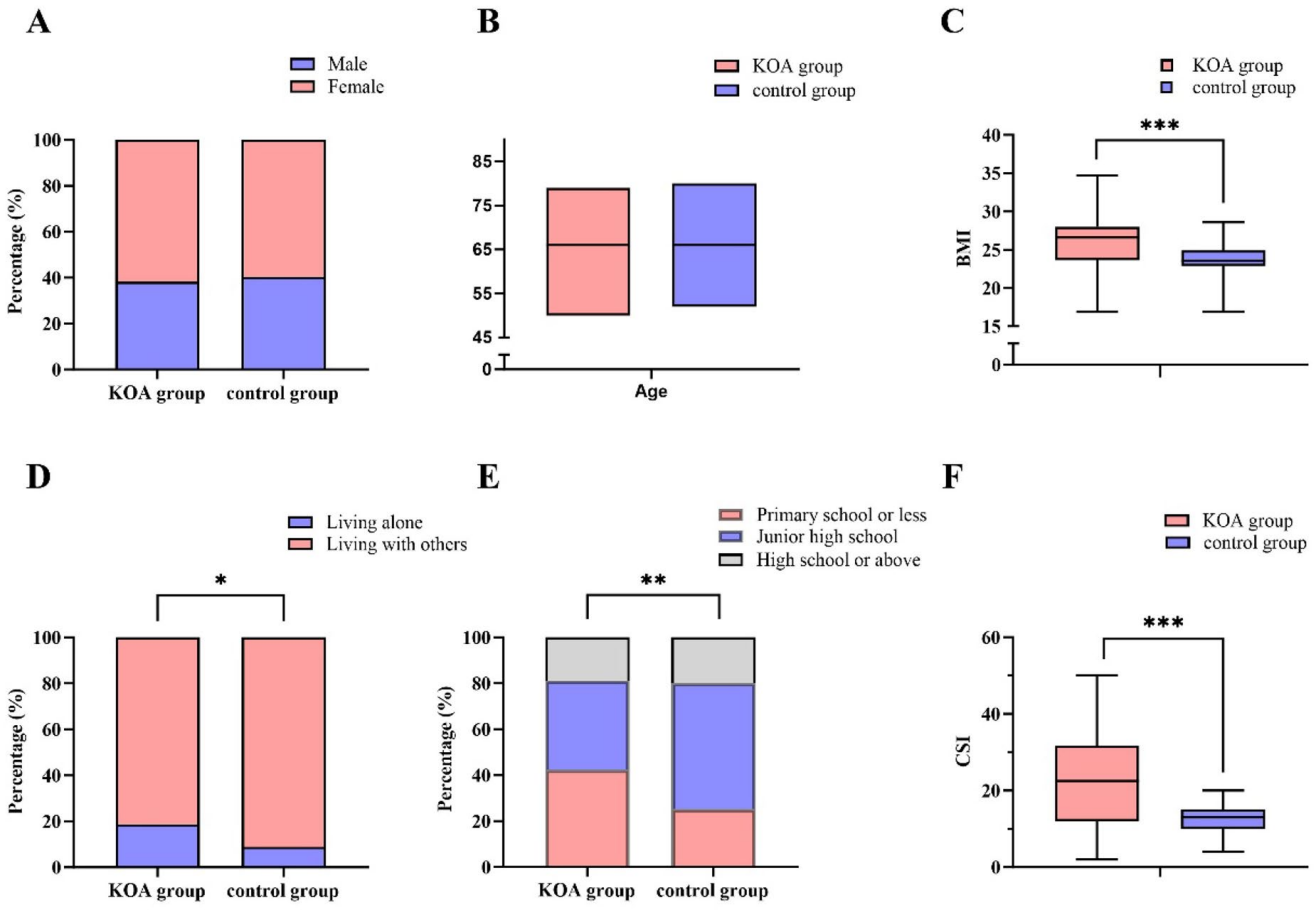
Comparison of baseline characteristics between KOA and control groups. (A) Gender distribution; (B) Age distribution; (C) BMI distribution; (D) Living status; (E) Educational level; (F): CSI score. *Note:* Continuous variables are presented as median (IQR), compared using Mann–Whitney U test; categorical variables as counts (%), compared using χ² test. *p < 0.05, **p < 0.01, ***p < 0.001.

The KOA group (*n* = 165) and healthy controls (*n* = 146) did not differ significantly in age or sex distribution (both *p* > 0.05), indicating good baseline matching. Specifically, males comprised 38.2% of the KOA group and 40.4% of controls (χ^2^ = 0.081, *p* = 0.775; Figure 2A), and the median age was 66 years (IQR: 61–71) in both groups (Z = −0.002, *p* = 0.998; Figure 2B).

In contrast, the KOA group had significantly higher median BMI compared with controls (26.6 kg/m^2^ [IQR: 23.7–28.0] vs. 23.6 kg/m^2^ [IQR: 22.9–24.9], *p* < 0.001; Figure 2C) and higher CSI scores (23 [IQR: 12–31] vs. 13 [IQR: 10–15], *p* < 0.001; Figure 2F). Moreover, the KOA group showed a higher proportion of participants living alone (18.8% vs. 8.9%, χ^2^ = 6.230, *p* = 0.013; Figure 2D) and with education level of primary school or below (42.4% vs. 25.3%, χ^2^ = 10.902, *p* = 0.004; Figure 2E).

### 3.2. Pressure pain threshold distribution and reference ranges

**Figure 3.**
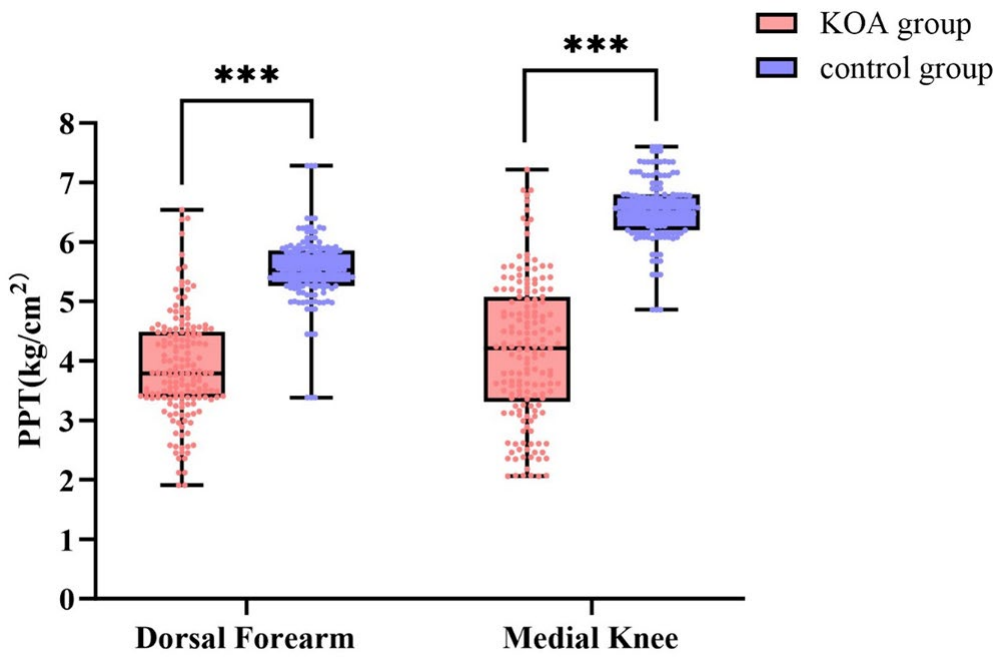
Distribution and 95% reference ranges of PPT at dorsal forearm and medial knee. *Note:* Red boxes and scatter points: KOA group; Blue boxes and scatter points: Control group. Box plots display the median, interquartile range (IQR) and extreme values of PPT, with individual data points overlaid to visualize the full data distribution; *** indicates a highly statistically significant difference between the KOA group and the control group (*p* < 0.001); unit of PPT: kg/cm².

Median PPT values at both the dorsal forearm and medial knee were significantly lower in the KOA group than in controls (both *p* < 0.001). Moreover, 95% reference ranges (P_2_._5_–P_97_._5_) were broader and left-shifted in the KOA group, indicating increased pain sensitivity and greater inter-individual heterogeneity (Figure 3).

For the dorsal forearm, the median PPT was 3.79 kg·cm^−2^ (IQR: 3.38–4.49) with a 95% reference range of 2.16–6.09 in the KOA group; in controls, the median was 5.53 kg·cm^−2^ (IQR: 5.26–5.86) and the 95% reference range was 4.10–6.69 (Z = −13.347, *p* < 0.001).

For the medial knee, the median PPT was 4.21 kg·cm^−2^ (IQR: 3.31–5.08) with a 95% reference range of 2.07–6.78 in the KOA group; in controls, the median was 6.57 kg·cm^−2^ (IQR: 6.20–6.80) and the 95% reference range was 5.26–7.55 (Z = −14.152, *p* < 0.001).

### 3.3. Correlations between PPT and influencing factors

#### 3.3.1. Spearman’s correlation analysis

**Figure 4.**
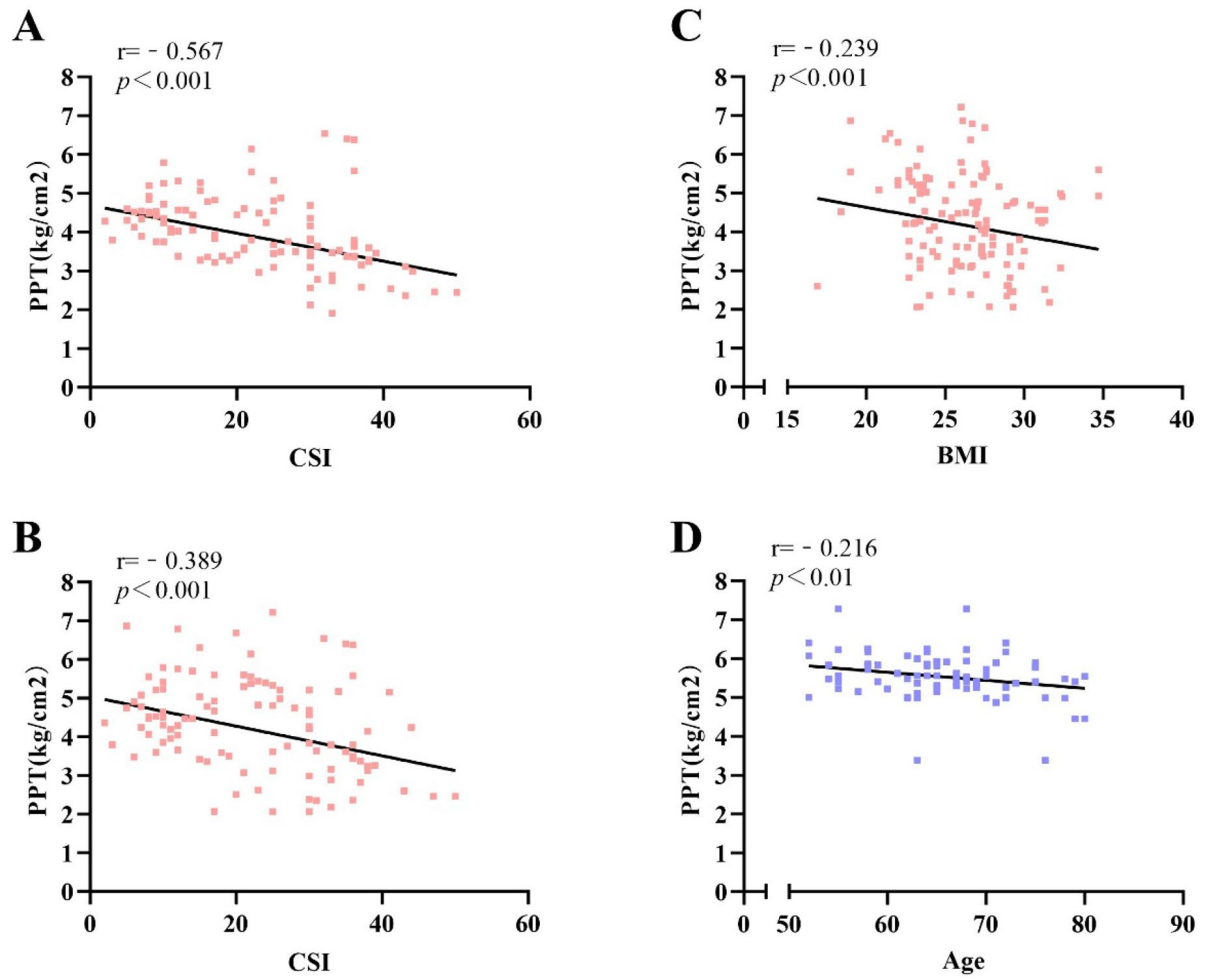
Scatter plots of PPT correlations. (A) Forearm PPT vs. CSI in KOA; (B) Knee PPT vs. CSI in KOA; (C) Knee PPT vs. BMI in KOA; (D) Forearm PPT vs. age in controls. *Note:* Red dots = KOA, blue dots = control; black lines = trend lines. Significant correlation defined as |r| ≥ 0.2, *p* < 0.05.

Correlations between PPT and variables differed between groups. In KOA patients, PPT was primarily associated with CSI scores and BMI, whereas in controls, only age correlated with forearm PPT (all *p* < 0.05, Figure 4).

In the KOA group, CSI score was moderately negatively correlated with forearm PPT (r = −0.567, *p* < 0.001) and weakly with knee PPT (r = −0.389, *p* < 0.001); BMI was weakly negatively correlated with knee PPT (r = −0.239, *p* < 0.001).

In the control group, only age was weakly negatively correlated with forearm PPT (r = −0.216, *p* = 0.012); no other significant correlations were observed.

#### 3.3.2. Intergroup and intragroup comparisons

Sex, living status, and educational level exhibited group-specific effects on PPT (Table 1).

**Table 1.** Intergroup comparisons of PPT across subgroups [median (IQR), kg·cm⁻²].

	KOA group (*n* = 165)		Control group (*n* = 146)	
Variables and subgroups	Forearm PPT	Medial knee joint PPT	Forearm PPT	Medial knee joint PPT
Gender				
Female	3.59 (3.37,4.30)	3.59 (3.13,4.78)	5.37 (5.15,5.53)	6.47 (6.15,6.68)
Male	4.25 (3.50,4.72)	4.71 (3.92,5.38)	5.83 (5.56,6.00)	6.74 (6.48,7.35)
*p* value	<0.001***	<0.001***	<0.001***	<0.001***
Living status				
Living alone	3.47 (2.99,4.25)	3.79 (2.61,4.67)	5.41 (5.00,5.70)	6.57 (6.15,6.77)
Non-living alone	3.80 (3.41,4.52)	4.24 (3.44,5.21)	5.53 (5.26,5.88)	6.57 (6.24,6.80)
*p* value	0.011*	0.026*	0.258	0.596
Educational level				
Primary school or below	3.49 (3.11,4.31)	3.80 (3.05,5.03)	5.40 (5.23,5.75)	6.59 (6.27,6.90)
Junior high school	3.80 (3.45,4.36)	4.20 (3.46,4.89)	5.56 (5.30,5.89)	6.57 (6.15,6.80)
High school or above	4.52 (3.60,4.93)	4.69 (4.06,5.55)	5.41 (5.26,5.82)	6.56 (6.36,6.73)
*p* value	<0.001***	0.017*	0.276	0.687

*Note:* Mann–Whitney U test for binary variables; Kruskal–Wallis H test for ternary variables, with Dunn’s post-hoc; *p < 0.05, **p < 0.01, ***p < 0.001.

In terms of sex, females had lower PPT than males in both groups (all *p* < 0.001). The difference in forearm PPT was 0.66 kg·cm^−2^ in the KOA group and 0.46 kg·cm^−2^ in the control group.

Regarding living status, non-living-alone participants in the KOA group had higher PPT than those living alone (forearm *p* = 0.011; knee *p* = 0.026), while no such differences were observed in the control group.

For educational level, only KOA patients showed significant differences: participants with high school education or above had higher PPT than those with primary school education or below (forearm *p* < 0.001; knee *p* = 0.017).

### 3.4. Hierarchical multiple linear regression of dorsal forearm PPT (KOA group)

**Figure 5.**
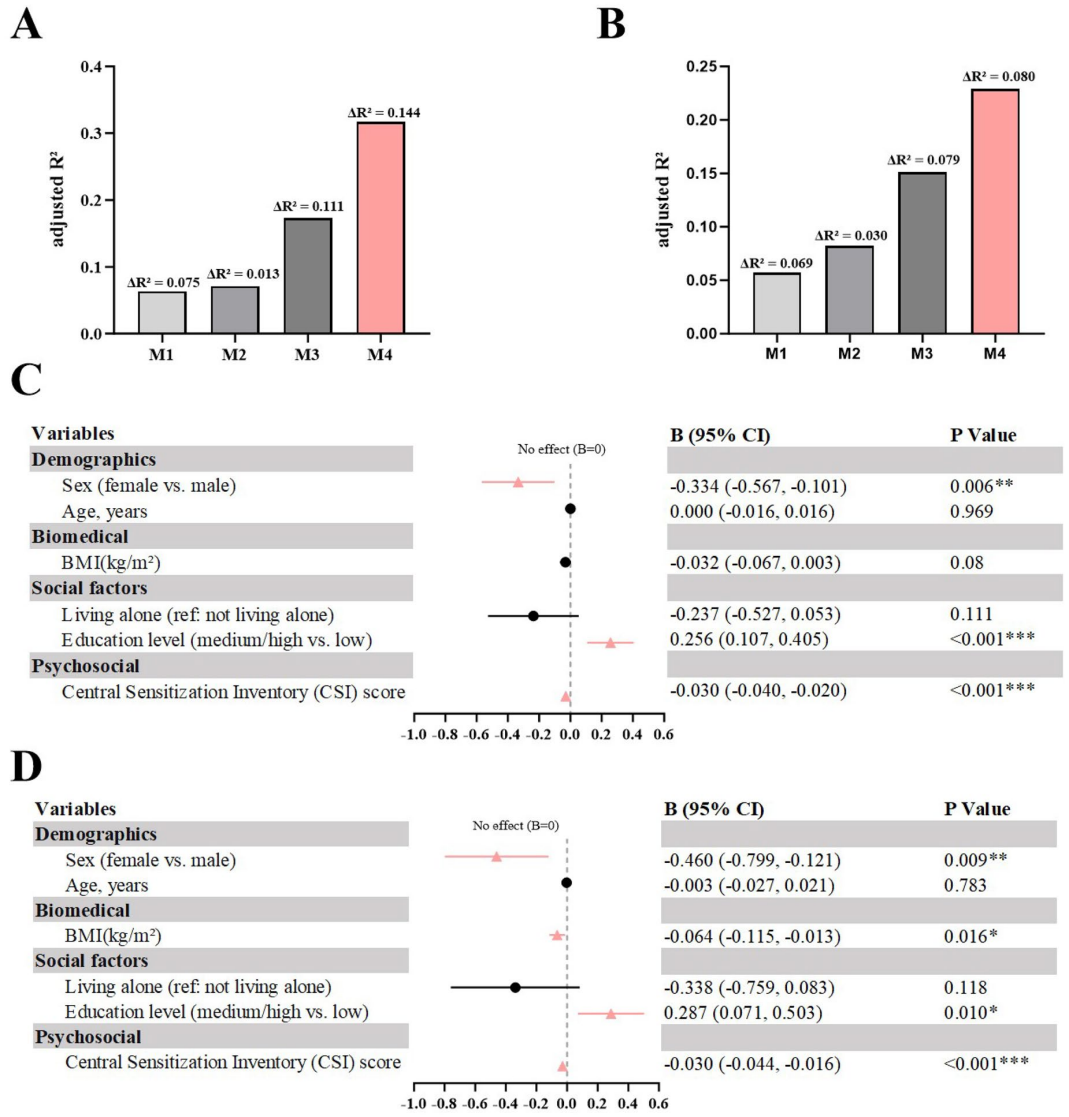
Hierarchical regression models and forest plots (KOA group). (A) Adjusted R² trend, dorsal forearm; (B) Adjusted R² trend, medial knee; (C) Forest plot of unstandardized regression coefficients (B) from the final model, dorsal forearm; (D) Forest plot of unstandardized regression coefficients (B) from the final model, medial knee. *Note:* Pink = significant predictors (*p* ≤ 0.05); horizontal lines = 95% CI; non-overlap with B = 0 indicates significance; *p < 0.05, **p < 0.01, ***p < 0.001.

Hierarchical block inclusion of variables progressively increased the explanatory power of the model, with CSI emerging as the key independent predictor (Figure 5A, 5C).

Model 1 (demographics) adjusted R^2^ = 0.063;
Model 2 (+ BMI) adjusted R^2^ = 0.071 (ΔR^2^ = 0.013, *p* = 0.135);
Model 3 (+ sociological factors) adjusted R^2^ = 0.173 (ΔR^2^ = 0.111, F = 10.980, *p* < 0.001);
Model 4 (+ CSI) adjusted R^2^ = 0.317 (ΔR^2^ = 0.144, F = 34.557, *p* < 0.001), representing a fourfold increase.

Independent predictors in Model 4: CSI (B = −0.030, 95% CI: −0.040 to −0.020, *p* < 0.001), educational level (*B* = 0.256, 95% CI: 0.107–0.405, *p* < 0.001), sex (B = −0.334, 95% CI: −0.567 to −0.101, *p* = 0.006). Living status became non-significant (*p* = 0.111). VIF < 1.13 indicated no multicollinearity.

### 3.5. Hierarchical multiple linear regression of medial knee PPT (KOA group)

Knee PPT was influenced by multiple factors, with higher explanatory power at all stages (Figure 5B, 5D).

Model 1 (demographics) adjusted R^2^ = 0.057;
Model 2 (+ BMI) adjusted R^2^ = 0.082 (ΔR^2^ = 0.030, F = 5.271, *p* = 0.023);
Model 3 (+ sociological) adjusted R^2^ = 0.151 (ΔR^2^ = 0.079, F = 7.624, *p* < 0.001);
Model 4 (+ CSI) adjusted R^2^ = 0.229 (ΔR^2^ = 0.080, F = 17.063, *p* < 0.001).

Independent predictors: CSI (B = −0.030, 95% CI: −0.044 to −0.016, *p* < 0.001), BMI (B = −0.064, 95% CI: −0.115 to −0.013, *p* = 0.016), sex (B = −0.460, 95% CI: −0.799 to −0.121, *p* = 0.009), educational level (*B* = 0.287, 95% CI: 0.071–0.503, *p* = 0.010). Living status non-significant (*p* = 0.118). VIF < 1.13.

### 3.6. Regression analysis of PPT (healthy controls)

**Figure 6.**
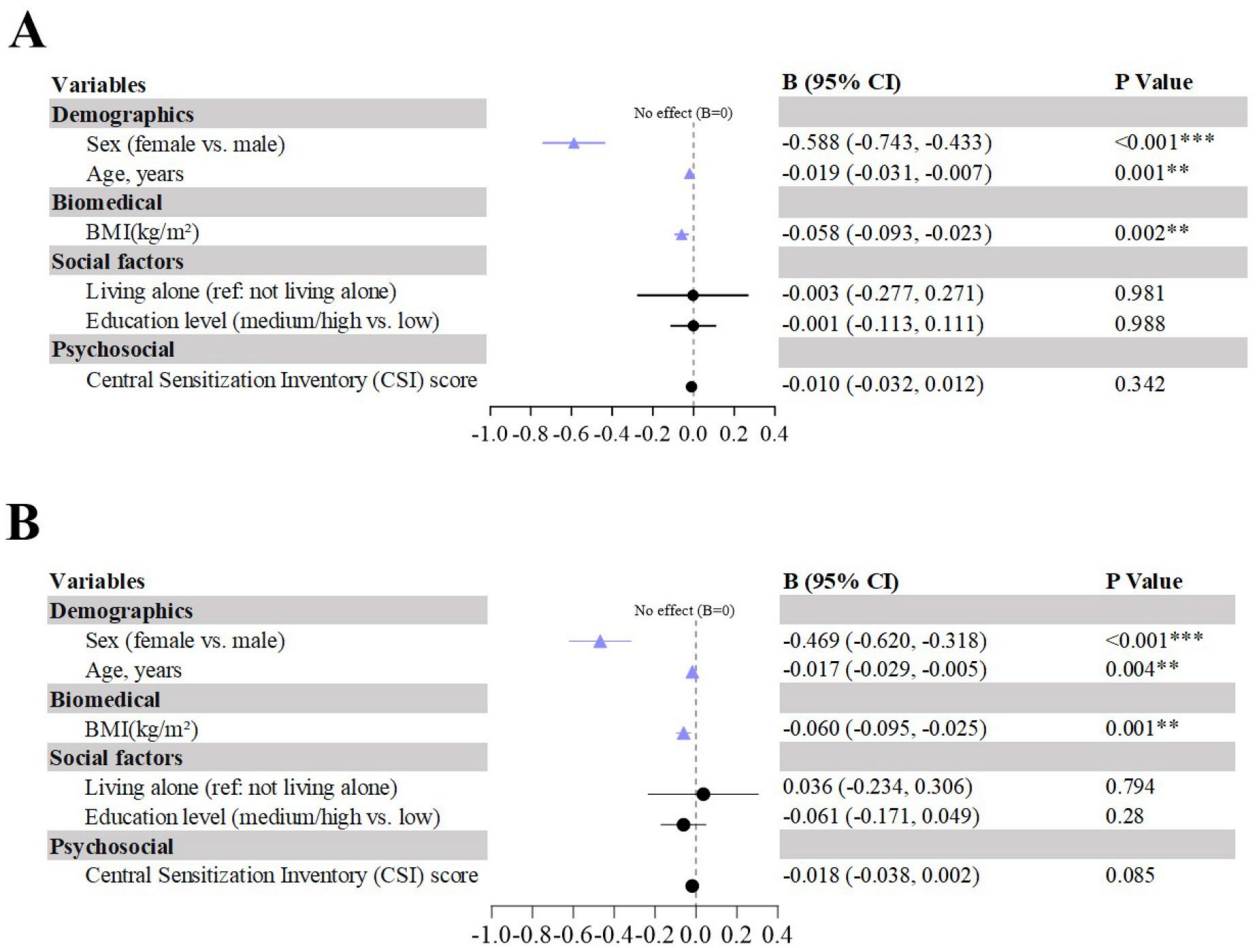
Forest plots of factors influencing PPT (healthy controls). (A) Forest plot of unstandardized regression coefficients (B) for forearm PPT; (B) Forest plot of unstandardized regression coefficients (B) for medial knee PPT. *Note:* Blue = significant predictors (*p* ≤ 0.05); all variables included simultaneously; *p < 0.05, **p < 0.01, ***p < 0.001.

Unlike the KOA group, CSI, educational level and living status did not predict PPT. Sex, age and BMI were core predictors (all *p* < 0.01, Figure 6).

For forearm PPT, the adjusted R^2^ was 0.332, with predictors including sex (B = −0.588, *p* < 0.001), age (B = −0.019, *p* = 0.001) and BMI (B = −0.058, *p* = 0.002).

For knee PPT, the adjusted R^2^ was 0.261, with predictors including sex (B = −0.469, *p* < 0.001), age (B = −0.017, *p* = 0.004) and BMI (B = −0.060, *p* = 0.001).

VIF < 1.10 indicated no multicollinearity.

## 4. Discussion

This study, for the first time, established preliminary percentile-based reference ranges for PPT at the knee joint and forearm in end-stage KOA patients in Xi’an, Northwest China. It identified generalized pain hypersensitivity as the predominant pain phenotype in this cohort, with the CSI score emerging as the strongest independent predictor of PPT. The demonstrated core correlation between CSI and PPT, together with the established reference ranges, provides objective evidence for developing a combined ‘CSI + PPT’ assessment system, optimizing KOA pain phenotyping and enabling precision analgesia, thereby filling a critical research gap in region-specific PPT reference data for KOA populations in Northwest China.

### 4.1. Generalized pain hypersensitivity as the core pain phenotype in end-stage KOA

By simultaneously measuring PPT at local (knee) and distal (forearm) sites, patients with end-stage KOA in this cohort exhibited significantly lower PPT values at both sites compared with healthy controls. The left-shifted and widened PPT reference ranges in the KOA group indicate the presence of generalized pain hypersensitivity linking local and systemic mechanisms [37]. This finding aligns with international studies; a meta-analysis of 1003 OA patients confirmed significantly reduced distal PPT, with hypersensitivity positively correlated with pain duration, highlighting the role of CS in chronic pain progression [31].

Notably, individual heterogeneity in PPT was substantial: despite similar radiographic severity, forearm PPT ranged from 1.91 to 6.54 kg·cm^−2^, with a more than 3-fold difference between the minimum and maximum values [38]. This observed heterogeneity can be partially explained by previous reports suggesting that a proportion of severe KOA patients exhibit features related to CS^30^. However, as participants were not stratified by CS status (using a validated CSI threshold), the observed heterogeneity should be interpreted cautiously and does not directly demonstrate the presence of distinct CS–dominant subgroups in this cohort. The PPT reference ranges obtained in this study provide a quantitative foundation for identifying ‘high-risk hypersensitive individuals’ and may help explain persistent chronic pain following joint replacement in some patients.

### 4.2. Association between CS symptoms and pressure pain threshold: Gradient correlation between CSI scores and PPT values

Spearman correlation and hierarchical regression analyses clarified the CSI–PPT relationship in this cohort. CSI scores were moderately negatively correlated with forearm PPT (r = −0.567) and weakly with knee PPT (r = −0.389). Incorporating CSI into the regression models increased explanatory power for forearm and knee PPT by ΔR^2^ = 0.144 and 0.080, respectively (both *p* < 0.001), indicating a moderate independent explanatory effect of CSI on PPT variation.

The median CSI score of the included patients was 23 (interquartile range: 12–31), with only 14.5% of patients meeting the widely validated clinical cut-off value of 40 points for clinically relevant CS. This suggests that a substantial proportion of the cohort did not meet criteria for clinically relevant CS, and the observed associations primarily reflect the link between subclinical CS-related symptoms and pain hypersensitivity rather than a dominant mechanism.

It should be noted that the present study was cross-sectional in design; therefore, the temporal sequence between PPT and CSI cannot be determined. Although we modelled CSI as an independent variable and PPT as the dependent variable for statistical analysis, this does not imply a causal direction. Clinically, reduced PPT may appear earlier as a manifestation of peripheral hyperalgesia and may subsequently contribute to the development of CS over time [33]. Accordingly, the regression results should be interpreted as associations rather than directional effects.

The gradient – stronger correlation at the distal site – is likely because forearm PPT is less affected by local joint pathology, thus more accurately reflecting central processes such as dorsal horn hyperexcitability and impaired descending inhibition. Conversely, knee PPT is confounded by local tissue status, diluting associations [30,33,34,39]. These findings indicate that forearm PPT is a more suitable indicator of CS-related changes in KOA patients [40], while knee PPT primarily reflects local tissue influences.

The CSI–PPT correlation in this study exceeded that reported in a systematic review by Neblett et al. (r = −0.36), likely due to focus on end-stage KOA with prolonged peripheral nociceptive input and use of standardized PPT protocols (fixed pressure rate, simultaneous dual-site measurement) [18]. Mechanistically, diffuse pain symptoms assessed by CSI are linked to insula and anterior cingulate cortex overactivation [41], while reduced PPT reflects heightened central neuron excitability [42]. Together, these findings provide complementary subjective and objective evidence for the association between CS-related symptoms and pain hypersensitivity, without implying it is the core or dominant mechanism underlying pain in all end-stage KOA patients.

Physiological factors showed weak predictive effects: BMI independently and negatively affected knee PPT (B = −0.064, *p* = 0.016), likely *via* synergistic peripheral and central mechanisms. Peripherally, higher BMI increases mechanical loading, accelerating cartilage degradation and synovitis [43]; obesity-related pro-inflammatory cytokines (TNF-α, IL-6) enhance nociceptor activation [44]. Centrally, persistent peripheral input induces sensitization, with insulin resistance altering neurotransmitter balance and microglial/astrocyte activation triggering inflammatory cascades, amplifying pain transmission and reducing knee PPT [44–47]. Sex effects were partially mediated by CS, as inclusion of CSI reduced the regression coefficient from −0.495 to −0.334, supporting Mogil et al.’s observations [34,48–50].

### 4.3. Potential modulatory role of sociological factors

Higher educational levels and cohabitation were associated with higher PPT in KOA patients in this cohort, but these effects attenuated after including CSI, suggesting indirect modulation *via* CS [51]. Individuals with higher education may possess better pain cognition and coping strategies, reducing catastrophizing and central facilitation [52]. Similarly, living with others may enhance social support, lower stress-induced cortisol, and protect descending inhibitory pathways [53]. These findings support targeted pain cognitive education for patients with low education or living alone.

Notably, psychological factors including anxiety, depression and sleep quality are well-recognized modulators of pain perception and CS, and are also closely related to the sociological factors discussed above. These negative emotional states and sleep disturbance may enhance pain catastrophizing, facilitate CS, increase pain sensitivity and ultimately result in lower PPT values [54,55]. However, these variables were not systematically assessed or adjusted for in our multivariable regression analyses of PPT, which may have introduced residual confounding in the observed associations between sociological factors and PPT values. Future studies incorporating standardized psychological assessments are needed to further clarify the independent modulatory effects of sociological factors on pain sensitization in KOA patients.

### 4.4. Differential regulation of PPT in healthy and pathological states: Implications for clinical phenotyping

In healthy controls from this cohort, PPT was influenced only by sex, age and BMI (adjusted R^2^ = 0.332), with no contribution from CSI or sociological factors, consistent with Gatchel et al. indicating that basic physiological conditions primarily determine pain sensitivity in healthy individuals [56]. In contrast, CSI substantially increased model explanatory power in KOA patients in this cohort (ΔR^2^ = 0.144), delineating the ‘physiological–pathological’ regulation boundary. CS thus serves as a key node for progression from local to systemic pain, highlighting the potential of the ‘CSI + PPT’ assessment system for precise KOA pain phenotyping.

### 4.5. Clinical translational value and future directions

Approximately 20% of KOA patients experience persistent pain after TKA [57,58]. This study provides a preliminary foundation for preoperative stratification, potentially enabling identification of CS-dominant patients. Validated protocols could inform central-targeted interventions (transcutaneous electrical nerve stimulation [59], cognitive behavioural therapy [60] preoperatively to reduce postoperative pain, supporting individualized perioperative pain management.

Notably, although CSI was analyzed as a continuous variable in the present study, previous validation studies have shown that a CSI score ≥ 40 may indicate clinically relevant CS, with acceptable sensitivity and specificity in KOA populations [61,62]. Consistent with these findings, a minority of end-stage KOA patients in our cohort met this cut-off criterion (14.5%), indicating that clinically relevant CS was present only in a subset of patients. This threshold may provide a preliminary reference for identifying CS-dominant KOA patients in clinical practice. When combined with the PPT reference ranges derived in the present study, such a cut-off may help improve risk stratification before TKA and enhance the clinical applicability of the proposed ‘CSI + PPT’ assessment system. However, the optimal population-specific threshold for end-stage KOA remains uncertain and requires validation in large-scale multicentre prospective cohorts.

Future research should focus on:

1. Validating the predictive value of the ‘CSI + PPT’ criterion for 1-year postoperative pain using multicentre prospective cohorts and defining the optimal population-specific cut-off thresholds for end-stage KOA;
2. Establishing a dual-dimensional behavioural–imaging assessment system incorporating functional magnetic resonance imaging (fMRI);
3. Comparing the efficacy of central-targeted versus conventional analgesic interventions in randomized controlled trials (RCTs).

### 4.6. Limitations

This study has several limitations:

1. Cross-sectional design precludes assessment of dynamic CS progression; longitudinal follow-up is needed.
2. This was a single-centre study conducted in Xi’an, Northwest China, which may introduce selection bias and limit the generalizability of the findings. The PPT reference ranges derived in this study should therefore be considered preliminary for this regional cohort and require validation in future multicentre studies across diverse regions of China.
3. Psychological factors known to influence pain perception and CS (anxiety, depression and sleep quality), as well as opioid medication history, were not included in the multivariable regression analyses, which may have resulted in residual confounding.
4. PPT relies on subjective responses; integration with biomarkers (peripheral inflammatory factors) could improve objectivity.

## 5. Conclusions

This study established preliminary percentile-based PPT reference ranges at the knee and forearm for end-stage KOA patients in Xi’an, Northwest China, confirming prevalent generalized pain hypersensitivity with marked inter-individual heterogeneity in this cohort. CS appears to contribute substantially to PPT variation, with CSI scores showing moderate correlations – particularly at the distal forearm – and serving as independent predictors in this regional population. The CSI–PPT relationship and preliminary reference ranges provide a foundation for constructing a combined ‘CSI + PPT’ assessment system, facilitating preliminary identification of patients with high-risk hypersensitivity potentially related to CS and supporting mechanism-based phenotyping, preoperative risk stratification, and informing future individualized pain management strategies in KOA, thus offering preliminary clinical translational value that requires further multi-centre validation across China.

## Data Availability

All datasets generated or analyzed during the current study are available from the corresponding author upon reasonable request.
